# The spectrum of hypophosphatemia in internal medicine: lessons from a Greek population study

**DOI:** 10.1007/s40618-025-02558-9

**Published:** 2025-04-04

**Authors:** Efstathia Megapanou, Matilda Florentin, Fotios Barkas, Haralampos Milionis, Eleni Bairaktari, George Liamis

**Affiliations:** 1 Department of Internal Medicine, University Hospital of Ioannina, Ioannina, Greece; 2 Faculty of Medicine, School of Health Sciences, University of Ioannina, Ioannina, Greece; 3 Laboratory of Clinical Chemistry, Faculty of Medicine, School of Health Sciences, University of Ioannina, Ioannina, Greece

**Keywords:** Drugs, Hyponatremia, Hypophosphatemia, Respiratory alkalosis

## Abstract

**Background:**

Hypophosphatemia, a common but often overlooked electrolyte disorder in hospitalized patients, lacks routine monitoring. This study aimed to assess the incidence, causes and associated biochemical and clinical features, as well as concurrent electrolyte and acid-base disturbances and mortality in patients with hypophosphatemia in an internal medicine ward.

**Methods:**

We prospectively included adult patients who either presented with or developed hypophosphatemia (serum phosphate levels < 2.5 mg/dL or 0.81 mmol/L) during hospitalization.

**Results:**

Among 4,095 patients, 4.3% (*n* = 176) had or developed hypophosphatemia. Of those, 126 patients (71.6%) had hypophosphatemia on admission, while 50 patients (28.4%) developed hypophosphatemia during hospitalization. All but one patient exhibited mild (72.2%) or moderate (27.3%) hypophosphatemia with serum phosphate levels between 2 and 2.5 mg/dL (0.65–0.81 mmol/L) and 1–2 mg/dL (0.32–0.65 mmol/L), respectively. The most common potential causes of phosphate depletion were respiratory alkalosis, malnutrition, drugs, e.g. diuretics and corticosteroids, diabetes mellitus and secondary hyperparathyroidism, with most patients (75.6%) exhibiting more than one likely causes. 64.8% of patients had at least one concomitant electrolyte disorder, the most common being hypocalcemia (40.9%), hyponatremia (38.6%), hypomagnesemia (23.9%) and hypokalemia (22.7%). 77% of patients exhibited pure or mixed acid-base disorders, mainly respiratory alkalosis (48.3%). Mortality was markedly higher in hypophosphatemic patients compared to the overall hospitalized population (15.9% vs. 4.26%). Furthermore, 8.1% of discharged patients had died within a month. Patients who developed hypophosphatemia during hospitalization were older, with higher incidence of hyponatremia and mortality rate (16% versus 5.6%). They also had higher parathyroid hormone and lower vitamin D levels, compared with those with hypophosphatemia on admission.

**Conclusions:**

Hypophosphatemia in internal medicine patients is often multifactorial and may signal greater illness severity. The high prevalence of associated electrolyte and acid-base disturbances suggests shared underlying mechanisms.

## Introduction

Hypophosphatemia is a common electrolyte disorder, affecting approximately 2–5% of hospitalized patients, with incidence rates significantly higher in specific clinical settings or among certain patient populations [1]. Hypophosphatemia is well-documented in surgical and intensive care units (ICUs), yet limited data exist on its incidence and patient characteristics in internal medicine settings. The prevalence and etiologies of hypophosphatemia vary across patient populations, potentially reflecting different underlying pathophysiological mechanisms. Low serum phosphate levels may serve as an indicator of disease severity, with studies linking hypophosphatemia to prolonged hospital and ICU stays, as well as to increased all-cause mortality. Furthermore, additional research has consistently demonstrated an association between low phosphate levels and elevated mortality risk [2, 3].

Phosphate (PO_4_^3−^) is an essential structural element of cell membranes, nucleic acids, proteins, as well as bone and dental tissue. It plays a crucial role in fundamental biological processes, including energy production via ATP synthesis, regulation of gene expression, enzyme synthesis and activity, and modulation of signaling pathways. Additionally, phosphate contributes significantly to the maintenance of electrolyte and acid-base balance [1, 3–6]. Consequently, phosphate depletion can impact multiple organ systems, with the most clinically significant effects observed in neuromuscular and cardiovascular function due to disruptions in their physiological processes.

In this prospective study of patients presenting with or developing hypophosphatemia during hospitalization in an internal medicine clinic, we investigated the underlying etiologies of hypophosphatemia and characterized the associated clinical and biochemical profiles. Additionally, we identified coexisting electrolyte and acid-base disturbances within this patient cohort and analyzed the associated mortality rate.

## Subjects and methods

### Study population

Over a 2.5-year period, we conducted a prospective study on an unselected cohort of consecutive adult patients (aged > 18 years) who exhibited hypophosphatemia either at admission or during hospitalization. The study was performed in the Internal Medicine Department of the University Hospital of Ioannina. Patients were included based on serum phosphate levels < 2.5 mg/dL (0.81 mmol/L) confirmed by two consecutive measurements to rule out laboratory variability. Hypophosphatemia was further categorized as mild (2–2.5 mg/dL or 0.65–0.81 mmol/L), moderate (1–2 mg/dL or 0.32–0.65 mmol/L), or severe (< 1 mg/dL or 0.32 mmol/L).

For each admitted patient, a comprehensive medical history was obtained, focusing on presenting symptoms, current medications (including over-the-counter drugs, supplements, and herbal products), comorbidities, alcohol use, smoking status, dietary intake, and any gastrointestinal losses.

The patients’ nutritional status was evaluated with the Malnutrition Universal Screening Tool (MUST) score [7, 8]. Malnutrition was defined as high MUST Score (2 or more points).

All medical interventions administered during hospitalization—including fluid therapy, drug treatments, inhaled β2 agonists, and intravenous ferric carboxymaltose—were meticulously recorded.

Prior to any therapeutic intervention, venous blood were collected to measure a comprehensive panel of biochemical and hematological markers, including glucose, urea, creatinine, uric acid, sodium (Na^+^), chloride (Cl^−^), magnesium (Mg^2+^), phosphate (PO_4_^3−^), calcium (Ca^2+^), potassium (K^+^), total protein, albumin, liver function test, thyroid stimulating hormone (TSH), C-reactive protein (CRP), lipid profile, intact parathyroid hormone (iPTH) and 25-hydroxyvitamin D [25(OH)D]. In patients with diabetes mellitus (DM), glycated hemoglobin (Hemoglobin A1c; HbA1c) was measured as well.

Serum Ca^2+^ levels were adjusted for hypoalbuminemia by adding 0.8 mg/dL (0.2 mmol/L) to the total serum Ca^2+^ concentration for every 10 g/L decrease in serum albumin from the reference value of 40 g/L.

Standardized equations were employed to calculate the fractional excretion (Fe) of key electrolytes, urea, and uric acid. A fractional excretion of phosphate (FePO_4_) exceeding 5% was indicative of renal phosphate wasting, suggesting inappropriate phosphaturia [9].

Arterial blood gas (ABG) analysis and urinalysis, including urine microscopy, were performed for each patient. Additionally, a fresh urine sample was analyzed for urea, creatinine, uric acid, electrolytes, and protein levels. Renal function was evaluated using the Cockcroft-Gault equation, and the estimated glomerular filtration rate (eGFR) was recorded for all participants. The comprehensive metabolic panel, ABG analysis, and complete blood count were repeated every other day to monitor changes over time.

Patients were stratified into two groups based on the timing of hypophosphatemia onset: those presenting with hypophosphatemia on admission and those who developed it during hospitalization. Following discharge, study participants (or their caregivers) were contacted after one month to assess their post-hospitalization health status.

## Analytical methods

Laboratory determinations were carried out by automated chemical analysis in our laboratory using an AU5800 Clinical Chemistry analyzer (Beckman Coulter, Hamburg, Germany). Arterial blood pH and PCO_2_ were determined using a pH blood gas analyser: serum bicarbonate was calculated from blood pH and carbon dioxide tension according to the Henderson-Hasselbalch equation with an acidity exponent of 6.10 and solubility coefficient of 0.0301.

### Statistical analysis

The Kolmogorov-Smirnov test was applied to assess the normality of distribution for quantitative variables. Data with a normal distribution are presented as mean ± standard deviation (SD), whereas non-normally distributed variables are reported as median with interquartile range (IQR). Qualitative variables are described using cumulative (N) and relative (%) frequencies. Pearson’s χ² test or Fisher’s exact test was employed for comparisons of categorical variables. Quantitative variables between groups were compared using Student’s t-test for normally distributed data, or the Mann-Whitney U test for non-normally distributed data. Statistical significance was defined as a two-tailed P value < 0.05. Analyses were conducted using SPSS software, version 22.0.

## Results

### Patient characteristics

A total of 176 patients met the inclusion criteria for this study. The mean age was 70.1 ± 20.8 years, with a sex distribution of 52.8% male and 47.2% female. Of these, 126 patients (71.6%) presented with hypophosphatemia on admission, while 50 patients (28.4%) developed it during hospitalization, most commonly from the third day onward (54%). Hypophosphatemia severity was predominantly mild (72.2%, *n* = 127), followed by moderate (27.3%, *n* = 48), with only one case of severe hypophosphatemia (0.5%). Baseline anthropometric and biochemical characteristics are detailed in Table 1.

**Table 1:** Baseline anthropometric and laboratory parameters of the study population

	Total number of patients with hypophosphatemia	**Hypophosphatemia upon admission**	**Hypophosphatemia during hospitalization**
Sex (%)			
Male	52.8	60.3*	34.0
Female	47.2	39.7	66.0
Age, mean value (SD)	70.1 (20.8)	66 (21.7)*	80.5 (13.9)
Malnutrition (High risk MUST SCORE) (%)	34.1	30.2	44.0
***Serum values***			
Urea, (mg/dl) median value (IQR)	38.5 (27.5–57.0)	37 (26 ─ 48)	49.5 (35 ─ 82)*
Creatinine, mg/dl (median value, IQR)	0.9 (0.8–1.3)	0,9 (0,8 ─ 1,1)	1 (0,7 ─ 1,6)
eGFR, ml/min/1,73 m^2^ median value (IQR)	77.2 (55.1–92.5)	82.6 (61.3–94.5)	67.1 (43.6–83.4) *
Total protein, (g/dl) median value (IQR)	6.3 (5.7–6.7)	6.3 (5.7–6.7)	6.3 (5.6–6.6)
Albumin, (g/dl) median value (IQR)	3.5 (3.0–3.9)	3.5 (3.1–3.9) *	3.3 (2.6–3.7)
Uric Acid, (mg/dl) median value (IQR)	5.4 (4.2–6.7)	5.2 (4.1–6.6)	5.5 (4.4–7.5)
Sodium, mEq/l median value (IQR)	137.0 (135.0–139.0)	138 (136 ─ 139)*	136 (133–139)
Potassium, mEq/l median value (IQR)	4.0 (3.6–4.3)	4 (3.7–4.4)	4 (3.5–4.2)
Calcium, mg/dl median value (IQR)	9.2 (8.8–9.6)	8.8 (8.4–9.4)*	8.4 (7.8–8.1)
Magnesium, mEq/l median value (IQR)	1.6 (1.4–1.8)	1.6 (1.4 ─ 1.7)	1.6 (1.4 ─ 1.8)
Chloride, mEq/l median value (IQR)	104.0 (100.0–107.0)	104 (101–106)	104.5 (98–107)
Hyponatremia (Na < 136 mEq/l), %	38.6	31.7	56.0*
Hypernatremia (Na > 145 mEq/l), %	2.8	1.6	6
Hypokalemia (< 3.5 mEq/l), %	22.7	19	30
Hyperkalemia (> 5.3 mEq/l), %	6.8	7.1	6
Hypocalcemia (Corrected Ca < 8.2 mg/dl), %	40.9	35.2	54
Hypercalcemia (> 10.6 mg/dl), %	3.4	4.0	2.0
Hypomagnesemia (< 1.3 mEq/l), %	23.9	24.6*	22
Hypermagnesemia (> 2.1 mEq/l), %	4.0	3.2	6.0
Hemoglobin, (g/dl) median value (IQR)	12.4 (10.8–13.8)	12.6 (10.8 ─ 13.9)	11.6 (10.2 ─ 13.3)*
White blood cells, (/µl) median value (IQR)	8750.0 (6630.0–11910.0)	8905 (6630 ─ 11950)	8350 (6500 ─ 11790)
Platelets, (/µl) median value (IQR)	199,000 (160000–252500)	203,500 (165000 ─ 254000)	189,000 (132000 ─ 229000)*
Ferritin, (ng/ml) median value (IQR)	203.5 (89.0–395.5)	163.5 (80 ─ 328) *	298 (139 ─ 605)
Hba1c, (%) median value (IQR)	6.1 (5.4–6.9)	6 (5.4 ─ 6.5) *	6,5 (5,7 ─ 7,6)
iPTH, (pg/ml) median value (IQR)	66.2 (36.3–102.5)	60 (36 ─ 100) *	130 (72 ─ 211)
VitD, (ng/ml) median value (IQR)	12.4 (9.8–18.6)	12.9 (10 ─ 19.3) *	7 (6.2 ─ 7.8)
FePO_4_, (%) median value (IQR)	18,8 (10,9–29,9)	17,6 (10,6–26,7)	19,4 (11,1–38,6)

*p < 0.05 for comparing patients with out-of-hospital and in-hospital hypophosphatemia (all levels of severity)

The incidence of hypophosphatemia among the 4.095 patients admitted during the study period was 4.3%. The mortality rate in hypophosphatemic patients was 15.9%, compared to 4.26% in the overall hospitalized population, and was notably higher in those developing hypophosphatemia during their hospital stay (16% vs. 5.6%). Infections were the leading cause of death, with an additional 8.1% of discharged patients dying within one month. Common reasons for admission included infections (29.5%), gastrointestinal bleeding (12.5%), stroke (8.9%), and diarrhea (8.9%), with no significant difference between groups.

Primary contributors to hypophosphatemia were respiratory alkalosis (48.3%), diuretic use (38.6%), malnutrition (34.1%), corticosteroid therapy (27.8%), and DM (25%). The presence of DM was not significantly linked to hypophosphatemia severity, and in 75.6% of cases, multiple factors likely contributed to phosphate depletion. In 8% of cases, no apparent cause was identified (see Table 2).

**Table 2:** Potential causes of hypophosphatemia

Causes of hypophosphatemia		(%)
Respiratory alkalosis (primary/mixed disorder)		48.3
Malnutrition		34.1
Diabetes Mellitus		25.0
Secondary hyperparathyroidism		18.8
Drugs	Diuretics	38.6
	• Loop diuretics	23.9
	• Hydrochlorothiazide and hydrochlorothiazide-like diuretics	13.6
	• Potassium sparing diuretics	1.1
	Corticosteroids	27.8
	inh. β2 agonists	18.8
	Anticonvulsants	16.5
	• Phenytoin	10.8
	• Phenobarbital	3.4
	• Valproic acid	1.1
	• Other	1.1
	Insulin	14.8
	Aminoglycosides	11.9
	Folic acid	9.7
	iv.Ferric carboxymaltose	9.1
Acute Tubular Necrosis		15.9
Diarrheal syndrome		11.9
Alcohol consumption		10.8
IV Glucose		9.1
Fanconi syndrome		1.1

Median fractional excretion of phosphate (FePO_4_ ^−3^) was 18.8%, with values < 5% in 7.5% of patients, 5–20% in 47.4%, and > 20% in 45.1%. Comorbidities included hypertension (54.5%), osteoporosis (35.8%), dyslipidemia (31.3%), peripheral artery disease (26.7%), stroke (26.1%), arrhythmias (26.1%), coronary artery disease (18.2%), heart failure (14.8%), cancer (14.8%), chronic obstructive pulmonary disease (13.6%), chronic kidney disease (10.8%), and excessive alcohol use (10.8%).

### Comparison between groups in baseline characteristics

Patients presenting with hypophosphatemia on admission were significantly younger (mean age 66 years) than those who developed hypophosphatemia during hospitalization (mean age 80.5 years), with a higher proportion of men (60.3% vs. 34%). This group also showed a trend toward better nutritional status, as indicated by the Malnutrition Universal Screening Tool (MUST) score, though the difference was not statistically significant (44%).

In contrast, patients who developed hypophosphatemia during their hospital stay exhibited lower estimated glomerular filtration rate (eGFR), albumin, hemoglobin, and platelet levels, alongside elevated ferritin levels compared to those with hypophosphatemia at admission (Table 1). Additionally, they had higher intact parathyroid hormone (iPTH) levels and lower vitamin D concentrations, suggesting a more pronounced disruption in calcium-phosphate homeostasis.

## Concomitant electrolyte disorders

A total of 64.8% of hypophosphatemic patients exhibited at least one additional electrolyte disorder. The most frequent abnormalities were hypocalcemia (40.9%), hyponatremia (38.6%), hypomagnesemia (23.9%), and hypokalemia (22.7%), while hyperkalemia (6.8%), hypermagnesemia (4%), hypercalcemia (3.4%), and hypernatremia (2.8%) were less common. The incidence of concurrent hyponatremia was significantly higher in patients who developed hypophosphatemia during hospitalization compared to those with hypophosphatemia on admission (56% vs. 31.7%).

Hypocalcemia, the most prevalent concurrent electrolyte disorder, was accompanied by elevated intact parathyroid hormone (iPTH) and low 25-hydroxyvitamin D levels in 18.8% of hypophosphatemic patients, indicating secondary hyperparathyroidism.

Serum phosphate levels were positively correlated with potassium (*r* = 0.138, *p* = 0.021), magnesium (*r* = 0.144, *p* = 0.016), and calcium (*r* = 0.178, *p* = 0.003), but not with sodium.

## Concomitant acid-base disturbances

Acid-base disturbances were identified in 77% of patients with phosphate depletion, primarily respiratory alkalosis, which affected 48.3% of the cohort. Respiratory alkalosis was either primary (38.2%) or part of mixed disorders (26.7%), including mixed metabolic and respiratory alkalosis (16%) or combined metabolic acidosis and respiratory alkalosis (10.7%). Common etiologies of respiratory alkalosis included gram-negative bacteremia, pulmonary conditions (notably pneumonia and pulmonary embolism), congestive heart failure, and hyperventilation due to pain or anxiety.

Metabolic acidosis was present in 8.4% of patients, commonly linked to renal failure, lactic acidosis, diarrhea, and ketoacidosis. Other acid-base disturbances, such as metabolic alkalosis, respiratory acidosis, and mixed metabolic alkalosis with respiratory acidosis, were rare (1.5%, 1.5%, and 0.8%, respectively). Hypophosphatemia showed no significant association with pH levels, and there were no notable differences between patients with hypophosphatemia on admission and those who developed it during hospitalization regarding the incidence of acid-base disorders.

## Discussion

Hypophosphatemia is a common electrolyte disturbance in hospitalized patients, with incidence rates varying significantly based on patient demographics and clinical conditions [2, 10–12]. In this study, the incidence of hypophosphatemia was 4.3%. Prior research has documented rates of 2.2–3.3% in general hospital populations, with severe cases comprising only 0.2–0.4%, findings [12–15], that align with our results. However, in critical care environments, hypophosphatemia is markedly more prevalent, occurring in 40–80% of ICU patients due to the heightened metabolic stress and complex comorbidities seen in these settings [11, 16, 17].

The prevalence of hypophosphatemia reported in the literature exhibits significant variability due to several factors, including incomplete data reporting from ICU patients, the infrequent routine measurement of phosphate levels, and the use of differing thresholds for defining low phosphate concentrations [2]. Notably, the clinical manifestations of hypophosphatemia do not consistently correlate with its severity [16], as phosphate primarily exists in intracellular compartments and does not accurately reflect total body stores. Consequently, this electrolyte disorder may often go undetected and remain asymptomatic [18]. Respiratory and muscular symptoms typically manifest when phosphate levels fall below 0.5 mmol/L, whereas neurological and cardiovascular symptoms are observed at levels below 0.3 mmol/L [1, 6, 10, 19].

Regarding the timing of hypophosphatemia onset, the majority of patients (71.6%) presented with hypophosphatemia upon admission, while 28.4% developed it during hospitalization. The incidence of in-hospital hypophosphatemia (1.3%) was relatively low compared to previous studies [20, 21]. The average hospital stay in our cohort was 3.5 days, which may have contributed to the lower incidence, as phosphate depletion typically manifests later in the course of illness when medical interventions, such as insulin or fluid administration, influence phosphate metabolism. Prolonged hospitalization may be associated with more severe illness and an increased risk of malnutrition, both of which could facilitate the development of hypophosphatemia. Indeed, the duration of hospital stay has been identified as a predictor of hypophosphatemia in univariate analyses of ICU patients [2, 11, 22, 23].

Hypophosphatemia predominantly affected elderly individuals in our study, with a mean age of 70.1 years; notably, 71% of participants were over 65 years old. Patients who developed hypophosphatemia during hospitalization were significantly older than those who presented with it at admission (80.5 vs. 66 years). This older cohort exhibited a higher prevalence of comorbidities, including osteoporosis, heart failure, atrial fibrillation, hypertension, chronic kidney disease, and atherosclerotic cardiovascular disease, consistent with findings from previous studies [20].

Patients who developed hypophosphatemia during hospitalization were older, exhibited poorer nutritional status, had more comorbidities, lower albumin levels, worse renal function, more severe anemia, and elevated ferritin levels. These findings indicate that this cohort was more severely ill and frail, which likely contributed to their higher mortality rate. Thus, hypophosphatemia may serve as a prognostic indicator of disease severity in this context.

The primary causes of hypophosphatemia included respiratory alkalosis (either primary or mixed), medication use, malnutrition, and DM. Notably, multiple factors contributed to hypophosphatemia in most patients, while a minority exhibited no discernible cause for their electrolyte disorder. These results align with previous studies that identified respiratory alkalosis, insulin, diuretics, glucose-containing solutions, and malnutrition as common contributors to decreased phosphate levels [24–27]. The complex etiology and multiplicity of potential causes have also been documented in the literature [28].

The traditional cut-off value for fractional excretion of phosphate (FePO_4_) indicating phosphaturia is 20%^30^. However, a threshold of 5% has been proposed for patients with hypophosphatemia, as the normal renal response to phosphate depletion is to enhance phosphate reabsorption, minimizing urinary phosphate excretion. Therefore, a FePO_4_ greater than 5% signifies renal phosphate wasting (inappropriate phosphaturia) in individuals with low serum phosphate [9].

In our study, 92.5% of patients exhibited inappropriate phosphaturia (FePO_4_ > 5%), underscoring the significant role of renal phosphate loss in the development of hypophosphatemia. The primary causes associated with inappropriate phosphaturia included drug use (notably diuretics), secondary hyperparathyroidism due to vitamin D deficiency, concurrent electrolyte disorders such as hypokalemia, acute tubular necrosis, and osmotic diuresis.

Respiratory alkalosis leads to hypophosphatemia by redistributing phosphate within cells [29], while metabolic acidosis (which was also common in our study population) promotes phosphaturia. Thus, these two acid-base disorders may be etiologically related to hypophosphatemia and their combination may further aggravate phosphate depletion.

Regarding malnutrition, poor intake alone is rarely responsible for severe phosphate depletion due to rapid renal adaptation; in fact, renal tubular phosphate reabsorption comes close to 100%, and, subsequently, urinary phosphate excretion approaches zero [30, 31]. Moreover, starvation alone does not lead to hypophosphatemia, as there is lack of insulin and increased cell catabolism which releases phosphate from cells. Importantly, though, the refeeding of malnourished patients may cause hypophosphatemia if no phosphate is provided. Therefore, other factors may have contributed to the development of hypophosphatemia even in patients with high MUST score. On the other hand, hypophosphatemia in elderly hospitalized patients has been associated with malnutrition and has been proposed as an indicator of poor nutrition; of course, normophosphatemia does not eliminate nutritional risk [32].

DM may also lead to hypophosphatemia via several mechanisms, e.g. osmotic diuresis (in inadequate glucose control), treatment with insulin or sodium-glucose cotransporter-2 (SGLT2) inhibitors, malabsorption and reduced vitamin D levels [33–37]. Moreover, diabetic patients frequently have hypertension and may take diuretics. Based on HbA1c levels (6% and 6.5% in the two study groups), most patients in our study had sufficient glycemic control; therefore, DM may have contributed to hypophosphatemia without being the only culprit. In few patients who were admitted with very high glucose levels (i.e. patients with ketoacidosis or uncontrolled DM which was further aggravated by an acute disease) hypophosphatemia was actually attributed to DM. Furthermore, the presence of DM did not differ between groups nor was DM associated with the severity of hypophosphatemia.

Drug-induced hypophosphatemia is frequently encountered in hospitalized patients. Several medications may lower phosphate levels through several mechanisms which have been previously described [38]. Among them, diuretics, corticosteroids, aminoglycosides, insulin and β2 agonists are the most common culprits [38–46].

We also showed that more than 3 out of 4 patients had at least one acid-base disorder, the most frequent being primary respiratory alkalosis. Mixed metabolic and respiratory alkalosis and mixed metabolic acidosis and respiratory alkalosis were also common. The mechanisms by which these acid-base disorders cause hypophosphatemia have already been discussed.

Regarding concomitant electrolyte disorders, hypocalcemia (40.9%), hyponatremia (38.6%), hypomagnesemia (23.9%) and hypokalemia (21%) were most frequently encountered in the study population. It is well known that in-hospital hyponatremia is much more common than out-of-hospital hyponatremia and that this electrolyte disorder is among the most common ones in internal medicine departments [47–49]. Only the incidence of hyponatremia differed significantly between groups, being higher in patients who developed hypophosphatemia in hospital. However, there was no correlation between sodium and phosphate levels in either group. The simultaneous presence of hyponatremia and hypophosphatemia may be attributed to common pathogenetic mechanisms and comorbidities associated with these disorders; the absence of correlation between these disorders further supports this hypothesis. Similarly, we have previously shown that among 204 patients with hyponatremia, hypophosphatemia was the most common coexisting electrolyte disorder (17%), mainly in the context of thiazide diuretic administration and syndrome of inappropriate antidiuretic hormone secretion, which causes phosphaturia through extracellular volume expansion [50].

The presence of hypocalcemia must be interpreted along with the elevated iPTH and reduced vitamin D levels. Vitamin D deficiency increases PTH both by reducing the intestinal absorption of Ca²⁺ and, thus, causing hypocalcemia and, to a lesser extent because the inhibitory action of calcitriol on PTH production is lost. In case of low vitamin D levels, the intestinal absorption of PO₄³⁻ is also decreased. Elevated PTH leads to phosphaturia and hypophosphatemia [51]. Therefore, low levels of 25(OH)D in a patient with hypocalcemia and hypophosphatemia suggest reduced intake and/or absorption of vitamin D, usually in combination with inadequate synthesis in the skin. Other possible causes include the administration of phenytoin, liver-biliary diseases, and nephrotic syndrome. Indeed, in our study, the most common cause of hypophosphatemia and hypocalcemia was secondary hyperparathyroidism due to low 25(OH)D levels, while several other diseases (e.g., pancreatitis, sepsis) and medications (e.g., bisphosphonates, loop diuretics, proton pump inhibitors, glucocorticoids) acted synergistically in a significant number of patients [52, 53]. We should note that the patients who developed hypophosphatemia in hospital were older and frailer and had lower vitamin D and higher iPTH levels compared with the other group.

Hypophosphatemia-related calciuria could also have contributed to the observed hypocalcemia taking into consideration that low serum phosphorus levels stimulate the production of 1,25-dihydroxy-vitamin D, which leads to an augmented intestinal calcium absorption and hypercalciuria [54, 55].

Furthermore, hypocalcemia could also be attributed to the coexistent hypomagnesemia (which is known to impair the release of PTH and induce skeletal resistance to its actions or/and hypokalemia which is associated with increased urinary excretion of calcium.

In the current study, serum phosphate correlated with serum potassium and serum magnesium levels, i.e. hypophosphatemia was significantly related to hypomagnesemia and hypokalemia.

The coexistence of hypophosphatemia and hypokalemia or/and hypomagnesemia is often attributed to common causative factors (e.g., diuretics, uncontrolled DM, respiratory alkalosis, acute tubular necrosis, malnutrition, increased gastrointestinal losses). Experimental and clinical observations have also demonstrated a close link among potassium, magnesium, and phosphorus concentrations. Indeed, potassium depletion is associated with increased urinary excretion of magnesium, calcium, and phosphorus, while magnesium depletion causes kaliuresis and potassium depletion). Moreover, magnesium depletion leads to renal phosphate wasting and phosphate depletion, although hypophosphatemia only rarely develops. Conversely, hypophosphatemia can cause inappropriate magnesiuria and hypomagnesemia.

Potassium levels have been found significantly decreased in ICU patients with hypophosphatemia compared with those without [11].

The mortality rate in our study population was significantly higher than that of the overall hospitalized patient population (15.9% vs. 4.26%) during the study period, with a greater prevalence among patients who developed hypophosphatemia during hospitalization compared to those with hypophosphatemia at admission.

Several studies have shown increased mortality rate in patients with hypophosphatemia mainly in ICU and in patients with sepsis [2, 21, 56]. Accordingly, patients with infections had the higher death rate in our study. It has been proposed that severe hypophosphatemia could be used as a mortality indicator, especially in patients with sepsis [3, 11, 23, 57, 58] as well as in patients with COVID-19 ^25^. Furthermore, ICU and hospital mortality may significantly increase in patients with hypophosphatemia [16]. In contrast, in other studies hypophosphatemia was not associated with increased mortality [4, 27, 59]. Overall, the role of hypophosphatemia in mortality has not been clearly elucidated. Probably this electrolyte disorder may serve as an indicator of poor outcome with different causative factors rather than a single independent etiology. In this setting, whether correcting hypophosphatemia is of clinical value is doubtful.

Data on post-discharge mortality is limited. In our study, 8.1% of discharged patients died within one month, indicating that hypophosphatemia may serve as a prognostic marker for poor outcomes in hospitalized patients. This association may be influenced by the presence of comorbidities and the effects of recent hospitalization on patient health [60–62].

### Study limitations and strengths

As an observational study, a key limitation was the inability to establish a definitive causal relationship between identified risk factors and hypophosphatemia. Additionally, the relatively small sample size and the single-center design limit the generalizability of our findings to broader patient populations. However, few studies have specifically focused on patients with hypophosphatemia, and many have not differentiated participants based on the timing of hypophosphatemia onset. Importantly, our study involved systematic measurement of phosphate levels in all admitted patients over a period of 2.5 years, allowing for a reliable estimation of hypophosphatemia incidence. Furthermore, the follow-up of study participants constitutes a significant strength of our research.

## Conclusions

In conclusion, our study provides valuable insights into the clinical implications of hypophosphatemia. This electrolyte disorder is typically multifactorial, with common contributing factors including respiratory alkalosis, various pharmacological agents, DM, and malnutrition. Furthermore, patients with hypophosphatemia often exhibit concomitant electrolyte and acid-base abnormalities. This condition is associated with increased in-hospital and short-term mortality; however, it remains to be determined whether low phosphate levels are a direct causative factor for adverse outcomes or simply a marker of underlying disease severity. Early detection of hypophosphatemia may be clinically significant, highlighting the need for further research to explore whether effective management of this disorder can enhance patient outcomes.
